# Electrochemically Synthesized Polyacrylamide Gel and Core–Shell Nanoparticles for 3D Cell Culture Formation

**DOI:** 10.1021/acsami.2c04904

**Published:** 2022-07-18

**Authors:** Nabila Yasmeen, Aneta Karpinska, Jakub Kalecki, Wlodzimierz Kutner, Karina Kwapiszewska, Piyush S. Sharma

**Affiliations:** † Institute of Physical Chemistry, Polish Academy of Sciences, Kasprzaka 44/52, 01-224 Warsaw, Poland; ‡ Faculty of Mathematics and Natural Sciences. School of Sciences, Cardinal Stefan Wyszynski University in Warsaw, Wóycickiego 1/3, 01-938 Warsaw, Poland

**Keywords:** electrochemically initiated persulfate decomposition, polyacrylamide gel, nanogel, core−shell nanogel, biocompatible gel, 3D cell culture

## Abstract

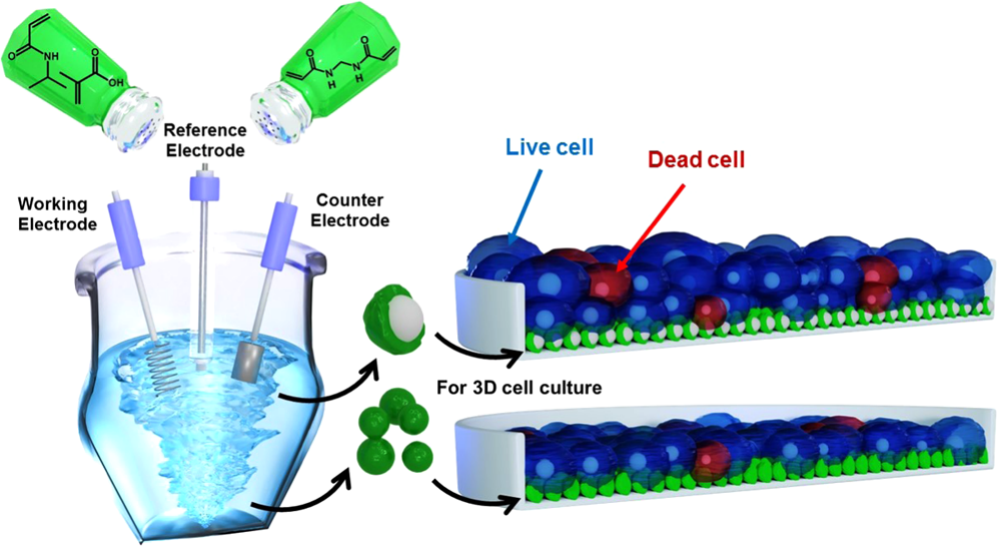

Biocompatible polyacrylamide gel and core–shell nanoparticles (NPs) were synthesized using a one-step electrochemically initiated gelation. Constant-potential electrochemical decomposing of ammonium persulfate initiated the copolymerization of *N*-isopropyl acrylamide, methacrylic acid, and *N*,*N*′-methylenebisacrylamide monomers. This decomposing potential and monomers’ concentrations were optimized to prepare gel NPs and thin gel film-grafted core–shell NPs. Scanning electron microscopy (SEM) and transmission electron microscopy (TEM) imaging confirmed the gel NP formation. The lyophilized gel NPs and core–shell NPs were applied to support the three-dimensional (3D) cell culture. In all, core–shell NPs provided superior support for complex 3D tissue structures.

## Introduction

The highly hierarchical nature of the human body is a spatially organized complex of multiple cell types. To better understand the cellular and noncellular environments, model systems derived from human cells are required.^1^ Animal models are inadequate because of the limited accessibility of imaging for observation, limited usability, ethical issues, and, most importantly, the vast differences between animal and human biology.^2^ Two-dimensional (2D) cell lines’ cultures, which are considered the simplest model, often lack cell–cell and cell–matrix interactions. These interactions are required to maintain and define in situ phenotypes. Therefore, 2D cultures fail to mimic cellular functions and signaling pathways present in tissues. Besides, when cultured in 2D, purified populations of primary cells can lose their phenotype.^3^ The aggregates of tumor cells, tissue slices, or spheroids may transiently capture the physiologically relevant cell–cell and cell–matrix interactions. Preferably, the development of culture systems will lead to relevant tissue organization and appropriate stem cell populations required in maintaining the three-dimensional (3D) culture systems for cell proliferation and differentiation.^4,5^

The cellular microenvironment of intrinsic ability is required to assemble complex cell structures as organ alternatives to fill the gap between the current model systems.^6^ This self-assembly of complex structures in 3D clusters is called organoids.^7^ Organoids are derived from embryonic stem cells, primary tissue-specific cells or induced pluripotent stem cells, and adult stem/progenitor cells.^8−10^ These spatially organized models are capable of self-renewal and self-organization.^11^ The physiologically relevant organoids help study organogenesis, disease modeling, and patient-specific therapies.^12^

Recent advances in micro/nanotechnology have driven organoid research progress.^3,13^ However, a high-quality microenvironment is still required to incorporate 3D structures into cell culture workflows. Moreover, physical parameters, including porosity, permeability, surface chemistry, and mechanical characteristics, are crucial for successful organoid growth.^14,15^

Organoid models offer unprecedented insight into human development processes and regenerative medicines, opening a new area of efficient human organ tissue formation and transplantation. Generally, the two categories of microenvironment workflows are used for organoid growth, the natural and synthetic hydrogel systems. The most commonly used microenvironment is Matrigel extracellular matrix (ECM). This medium provides a proper biological environment for cell survival and proliferation for organoid formation.^16,17^ Matrigel matrix or ECM-based basement membrane is extracted from Engelbreth–Holm–Swarm mouse tumors.^18^ These membranes are enriched with growth factors and compounds crucial for cell proliferation and differentiation, such as laminin, heparan sulfate proteoglycan, and nidogen/entactin.^19^ These components help activate signaling pathways to control angiogenesis, cancer cell motility, and drug sensitivity.^11,20^ However, natural animal-derived matrix systems have their limitations, as they are not well defined and always at a high risk of batch-to-batch variability.^21^ Therefore, synthetic polymer scaffolds are a promising alternative. These scaffolds can provide a pathogen-free, biologically inert, highly tunable microenvironment for the growth of the organoid system of choice.^22^ Commonly, synthetic hydrogel systems incorporate the signaling proteins, specific biofunctionalities, and growth arginine–glycine–aspartic acid (RGD) motifs to mimic the natural microenvironment.^23−25^ The most common organoid formation ways include bioprinting, soft lithography, micromolding, hanging drop procedure, magnetic levitation, and microfluidics.^11,26,27^

Synthetic polymer hydrogels are a promising tool in organoid formation due to tunable physicochemical properties, cross-linked 3D structures, and biocompatibility.^28,29^ Furthermore, hydrophilicity and porosity of polyacrylamides provide similarities to cellular microenvironments, such as Matrigel or ECM.^28,30−33^ Likewise, stiffness, stability, and cell adhesion (addition of RGD peptides) are vital biochemical parameters for tissue formations.^34−36^ Besides, the cross-linking and mechanical properties of the gels significantly influence cell adhesion, which, in turn, regulates cell proliferation and migration in the cellular microenvironment.^37^

Currently, the available gels’ biocompatibility is low and the mechanical stability is weak.^38^ These two factors are usually tuned by selecting the most suitable cross-linking monomers and adding functionalities using appropriate comonomers, synthetic solvents, or varying synthetic procedures.^39−42^ Noticeably, decreased mechanical strength can be considered an advantage because it helps retrieve the prepared tissues for further applications.^39^

Previously, we described copolymerization of a mixture of highly tunable amphiphilic *N*-isopropyl acrylamide **NIPAM**,^43−45^ the pH-responsive methacrylic acid **MA** functional monomer, and the *N*,*N*′-methylenebisacrylamide **BIS** cross-linking monomer in an aqueous solution to synthesize microsized particles.^46^ This polymerization was initiated electrochemically by applying suitable potential. Compared to other microgel preparation ways, this approach is simple to perform and control. Its main advantages include no need for additives, surfactants, and hazardous solvents. Moreover, polymerization can proceed at room temperature. Therefore, it can be referred to as the “green” synthesis. Solvent polarity played a significant role in determining the synthesized gel’s physicochemical and mechanical properties.^42,47^ Therefore, the electrochemical synthesis of gels was performed in an aqueous medium.^46^ Moreover, microgels were grafted on inorganic substrates with this unique approach to prepare core–shell particles at room temperature. Overall, this approach allowed the synthesis of microgel particles in solution but lacked control over gel size and morphology.

In the current work, electrosynthesis conditions are further optimized to improve the gel size. Toward that, the cross-linking monomer concentration and electroinitiation potential are optimized. Finally, the gels are used as a basement membrane for 3D cell culture formation using two different cancer cell lines.

## Experimental Section

### Chemicals

*N*-Isopropyl acrylamide **NIPAM**, methacrylic acid **MA**, *N*,*N*′-methylenebisacrylamide **BIS**, ammonium persulfate **APS**, and magnetic nanoparticles (MNPs) were purchased from Sigma-Aldrich. Potassium chloride (KCl) was from POCH, Poland. The 500 nm diameter silica beads were procured from Fiber Optic Centre. 3-(4,5-Dimethylthiazol-2-yl)-2,5-diphenyltetrazolium bromide, an **MTT** reagent, was ordered from Thermo Fisher Scientific. Deionized ultrapure Merck Millipore Milli-Q water (18.2 M Ω·cm) was used to prepare all aqueous solutions. All reagents and solvents were of analytical grade and used as received.

### Biological Materials

MDA-MB-231 and HeLa cell lines were ordered from the American Type Culture Collection (ATCC, Manassas). Both cell lines were cultured as a standard monolayer in the complete growth medium, supplemented with 10% *v*/*v* fetal bovine serum (FBS, Gibco), l-glutamine 1%, *v*/*v* (Sigma-Aldrich), and the antibiotics, namely, streptomycin (10 mg/mL) and penicillin (10,000 U/mL) 1%, *v*/*v* (Sigma-Aldrich). Cells were cultured in a 5% CO_2_ atmosphere at 37 °C. The HeLa cell line was cultured in Dulbecco’s modified Eagle’s medium (DMEM) with low glucose content (Institute of Immunology and Experimental Technology, Wrocław, Poland). The culture medium for MDA-MB-231 was RPMI-1640 with sodium bicarbonate and without l-glutamine (Sigma-Aldrich).

By regular passages, cells were maintained in a logarithmic growth phase. Cells were detached from the surface with a 0.25% trypsin–EDTA solution (Sigma-Aldrich). The trypsinization was controlled using light microscopy.

### Electrochemical Gel Particle Synthesis

For electrochemical gel preparation, an SP-300 BioLogic potentiostat was used. The potentiostat was controlled by EC-Lab BioLogic software. The gel particles were prepared after electrochemical initiation using a homemade electrochemical cell. Electrochemical experiments were carried out using a homemade conically shaped three-neck glass cell with a volume of ∼30 mL. A 250 μm diameter Pt coiled wire (∼20 cm), a Ag|AgCl electrode, and a stainless steel gauze electrode (3 cm × 3 cm) were used as the working, reference, and auxiliary electrodes, respectively.

The solution of 20 mM **MA**, 20 mM **NIPAM**, and 10 mM **BIS** was used for the polymerization. Initiator concentration was minimized to 15 mM **APS** in the polymerization solution. Before reaching this optimized monomer composition, other monomer molar ratios were employed to synthesize gel particles, and morphological features were visualized. A 0.1 M KNO_3_ supporting electrolyte was added to make the polymerization solution conductive. The solution was deoxygenated with a 20 min argon purge.

Furthermore, during all experiments, argon was continuously purged through the solution. During polymerization, the working electrode potential was kept constant at −0.60 V vs Ag quasi-reference electrode for 1–3 h depending on monomer composition under constant stirring. The gel particles were collected after 30 min of stopping the electrochemical initiation. Next, gel particles were centrifuged at 20,000 rpm for 20 min. This centrifugation was repeated three times for the complete removal of unreacted substrates.

For the electrosynthesis of the gel film-grafted MNPs, a 2.5 mL of 3 mg mL^–1^ dispersion of MNPs was added to a 25 mL sample of the solution for polymerization, which was 20 mM in **MA**, 20 mM in **NIPAM**, and 10 mM in **BIS**. First, silica particles were surface-functionalized with 3-(trimethoxysilyl)propyl methacrylate using an earlier reported procedure to prepare silica-based core–shell particles.^46^ A 0.5 mL sample of this suspension was added to the 20 mL sample of the solution for electrochemically aided polymerization. The core–shell particles were centrifuged at 20,000 rpm for 20 min. This centrifugation was repeated three times for the complete removal of unreacted substrates.

The pellet of the gel particles (with and without cores) was collected after centrifugation for lyophilization. The samples were first freeze-dried for 24 h and then evaporated in a vacuum for 3–4 h. Finally, the lyophilized gel particles were stored in airtight vials for further characterizations.

### Scanning Electron Microscopy (SEM) Gel Particle Imaging

Gels’ samples were imaged with scanning electron microscopy (SEM) using a Nova NanoSEM 450 microscope of the FEI Nova. Microgel samples were first dispersed in an aqueous solution and then drop-coated onto Au film layered glass slides for this imagining. The SEM images were taken with a 3–5 kV acceleration voltage and a working distance of 5 mm without coating the samples.

### Fourier Transform Infrared (FTIR) Spectroscopy Measurements

Gel sample infrared (IR) spectra were recorded with a Vertex 80v Fourier Transform IR (FTIR) spectroscopy using a one-reflection ATR computer-controlled Bruker spectrometer equipped with Opus 6.5 software of the same manufacturer. Spectra were recorded with a 2 cm^–1^ resolution. For each spectrum, 1024 scans were acquired. Measurements were performed under a decreased (6 hPa) pressure.

### Dynamic Light Scattering (DLS) Measurements

Dynamic light scattering (DLS) was used to measure the hydrodynamic particle diameter. This measurement was carried out using a Zetasizer NS instrument (Malvern Instruments, Ltd.). The microgel suspension (0.1%, *w*/*v*) was prepared using Milli-Q water (pH = 7.4) and then sonicated for 15 min before the DLS analysis. Concentration-dependent effects were avoided by preparing diluted suspensions of gel particles (0.1%, *w*/*v*). Each measurement was repeated three times, and the average particle size, *Z*_avg_, was reported.

### In Vitro Cytotoxicity Assays Involving Nanogels and Core–Shell Nanoparticles

Cell viability was checked through the MTT proliferation/(metabolic activity) assay. We performed this cytotoxicity assay for two different cancer cell lines, viz., MDA-MB-231 (triple-negative breast cancer) and HeLa (cervical cancer). Approximately 5000 cells/well for MDA-MB-231 and 10,000 cells/well for HeLa (controlled with Countess II Cell Counter) were seeded into a 96-well plate (Greiner Bio-One). Afterward, cells were incubated for 24 h at 37 °C in an incubator. Then, the medium was removed, and gel particles (dispersed in acidic, neutral, or alkaline solutions) at different concentrations (20,000–39 ng/mL) were added to the cell fresh medium. The experiments were repeated three times for each concentration. After 24 h, the medium containing tested gel particles was replaced with a culture medium, which was 1 mM in 3-(4,5-dimethylthiazol-2-yl)-2,5-diphenyltetrazolium bromide. Cells were incubated for 4 h at 37 °C. Then, the solutions were replaced with DMSO and incubated for another 10 min. The absorbance was measured at 540 nm using a Synergy HTX multimode reader (BioTek). We also performed controls: blank, medium without cells; positive control, cells not treated with gel particles; and negative control, dead cells, toxicant, 1% *v*/*v* Triton-X-100 (Sigma-Aldrich).

### 3D Cell Culture

MDA-MB-231 and HeLa cells were detached from culture flasks using trypsin–EDTA and resuspended in fresh culture media to a cell density of 10^6^ cells/mL. Next, 10 μL of the cell suspension (10^4^ cells) was placed in wells of 8-well CellVis plates. Cells were then covered with 330 μg/mL nanoparticles suspended in a buffer, filled with fresh medium up to 400 μL, and then placed for incubation. The pure cell suspension was placed in another well as a control. Half of the cell culture medium was replaced every second day in all wells. Three separate experiments were performed for 1, 2, and 3 week experiments.

### Confocal Microscopy Imaging

Cells were stained with calcein-AM (Merck) and propidium iodide (Merck) for confocal microscopy imaging. Calcein-AM is a green dye staining living cells only; it was added to the medium to reach a concentration of 1 μg/mL. Propidium iodide stains the nucleic acids red in dead cells; its concentration in the test solution was 0.1 mg/mL. Samples were incubated with the dyes for 20 min and then imaged. The samples were imaged using a Nikon A1 inverted confocal microscope (Nikon Instruments) coupled with NIS Elements software (Nikon Instruments). The signals from viable and dead cells were recorded using fluorescein isothiocyanate (FITC) and tetramethylrhodamine isothiocyanate (TRITC) settings, respectively. The 3D images were recorded using the *Z*-stack mode of NIS Elements software. Then, 3D images were further analyzed using Imaris software (Oxford Instruments).

Statistical analysis was performed using the Analysis ToolPak add-in to Microsoft Excel Software. The data sets were compared using a two-sample *t*-test assuming unequal variances. The analysis of 3D cell cultures was performed in two directions: (1) statistically significant difference from the control and (2) statistically significant differences between cell lines and sample preparation conditions within the same time points.

## Results and Discussion

Previously, we proposed an electrochemically initiated synthesis of polyacrylamide microgels.^46^ In this unique approach, microgels were prepared with and without core–shell support using amphiphilic and hydrophilic monomers. More importantly, this polymerization was initiated at room temperature. Overall, it led to microsized gel particles with irregular morphology. Therefore, further optimization was needed to improve the morphology and size of these particles. Uniform nanosized gel particles are best suited for biomedical applications.

### Electrochemically Initiated Synthesis of Gel Particles

Herein, we report the improvement of the gel particles’ architecture. Because of a relatively high monomer solubility, the solvent for gel synthesis was not changed, and an aqueous solution was chosen. The chain-transfer efficiency increased during aqueous polymerization because of the strong intermolecular hydrogen bonding between the monomers’ amide (O=C–NH_2_) groups and water molecules.

Later, another critical factor, i.e., the electroinitiation potential, was optimized. For that, potentials different from −0.60 V, i.e., −0.70 and −0.80 V vs Ag quasi-reference electrode, were applied for 3 h, while monomer concentrations were 25 mM **NIPAM**, 25 mM **MA**, and 50 mM **BIS**, as previously reported.^46^ When a more negative potential of −0.80 V was applied, the gel appearance in the solution was extended over 24 h. This time shortened when a potential of −0.70 V was selected. The shortest gel formation time was needed when a potential of −0.60 V was applied to break ammonium persulfate to generate free radicals in the solution.^46^

An alternative factor that controls the morphology of gel particles is monomers’ composition. Therefore, gels were synthesized using cross-linking monomers of different concentrations. The **BIS** cross-linking monomer concentration increase up to 50 mM in the polymerization solution nearly did not change the morphology of the resulting gel particles. However, the polymer morphology was substantially changed at concentrations exceeding 50 mM BIS.

Another polymerization solution of 20 mM **MA**, 20 mM **NIPAM**, and 10 mM **BIS** was used. In this polymerization, monomers’ concentrations were slightly lower than those reported previously.^46^ Surprisingly, under these monomers and cross-linking monomer concentration conditions, the gelation commenced after only 20 min of electroinitiation. Nevertheless, the potentiostatic condition was continued for 1 h. After completing this potentiostatic experiment, we collected the gel particles after 30 min by centrifuging and dispersing them again in water. This cycle was repeated three times to obliterate the unreacted substrates. In the Supporting Information, we discussed the mechanism occurring during the electrochemically initiated microgel synthesis (Scheme S1).^46,48^

Core particles were dispersed in a solution of monomers and cross-linking monomers of a composition similar to that described above to synthesize core–shell particles.

### Morphological Characterization of Gel Particles and Core–Shell Nanoparticles

The morphology of gel particles prepared at different electroinitiation potentials (Figure S1 in the Supporting Information) and different cross-linking monomer concentrations was examined by SEM imaging (Figure S2 in the Supporting Information). Gels prepared at lower potentials were separate particles, whereas gels prepared at higher potentials were more bulk-like (Figure S1 in the Supporting Information). Therefore, a lower electroinitiation potential was chosen for further study.

Figure S2 in the Supporting Information shows SEM images of **NIPAM–MA–BIS** gel particles synthesized using cross-linking monomers of different concentrations. The **BIS** cross-linking monomer concentration increase up to 50 mM in the polymerization solution nearly did not change the morphology of the resulting gel particles. However, this morphology was substantially changed at concentrations exceeding 50 mM **BIS** (e.g., 100 mM). More deformed particles were obtained after increasing the **BIS** concentration in the solution for polymerization (Figure S2 in the Supporting Information). An increased cross-linking monomer concentration decreased the gel porosity and affected its solubility.

This radical polymerization might not be sufficiently quenched after seizing the electrochemical reaction. Therefore, a polymerization inhibitor, viz., hydroquinone monomethyl ether, was added after stopping electroinitiation. Three different inhibitor concentrations were used, i.e., 5, 15, and 25 mM, to study the polymerization chain inhibition effect on the gel particles’ morphology (Figure S3 in the Supporting Information). Nevertheless, globular gel particles of different sizes were obtained in each case.

**Figure 1:**
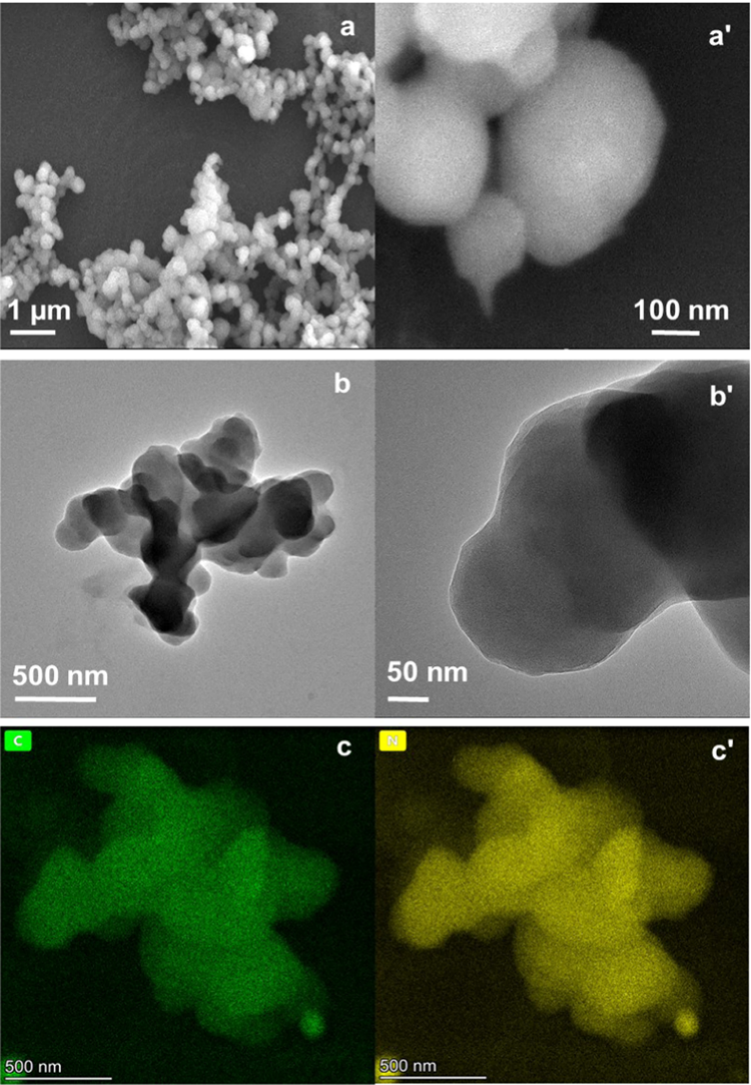
(a, a′) SEM and (b, b′) TEM images of the **NIPAM–MA–BIS** gels prepared at the monomers’ concentrations of 20, 20, and 10 mM, respectively. (c, c′) Mapping of the elemental distribution of the **NIPAM–MA–BIS** gel particle; carbon (green) and nitrogen (yellow) elements.

The SEM image of the solution for polymerization, which was 20 mM in **MA**, 20 mM in **NIPAM**, and 10 mM in **BIS**, justified the tuning of electropolymerization conditions (Figure 1). Decreasing the cross-linking monomer concentration resulted in the successful preparation of nanometer-sized gel particles (Figure 1a,a′). TEM images confirm the globular shape nanogel synthesis (Figure 1b,b′). These results suggest that the gel’s overall morphology depends on the cross-linking monomer concentration. In a way, a low cross-linking concentration can improve the swelling property, porosity, and mechanical stability of the gel nanoparticles prepared.^49^ The energy-dispersive X-ray (EDX) mapping indicates the elemental distribution of the **NIPAM–MA–BIS** gel nanoparticles (Figure 1c,c′). This mapping reveals that the particles were composed of carbon (green) and nitrogen (yellow) elements, and these elements were evenly distributed in the gel particles.

**Figure 2:**
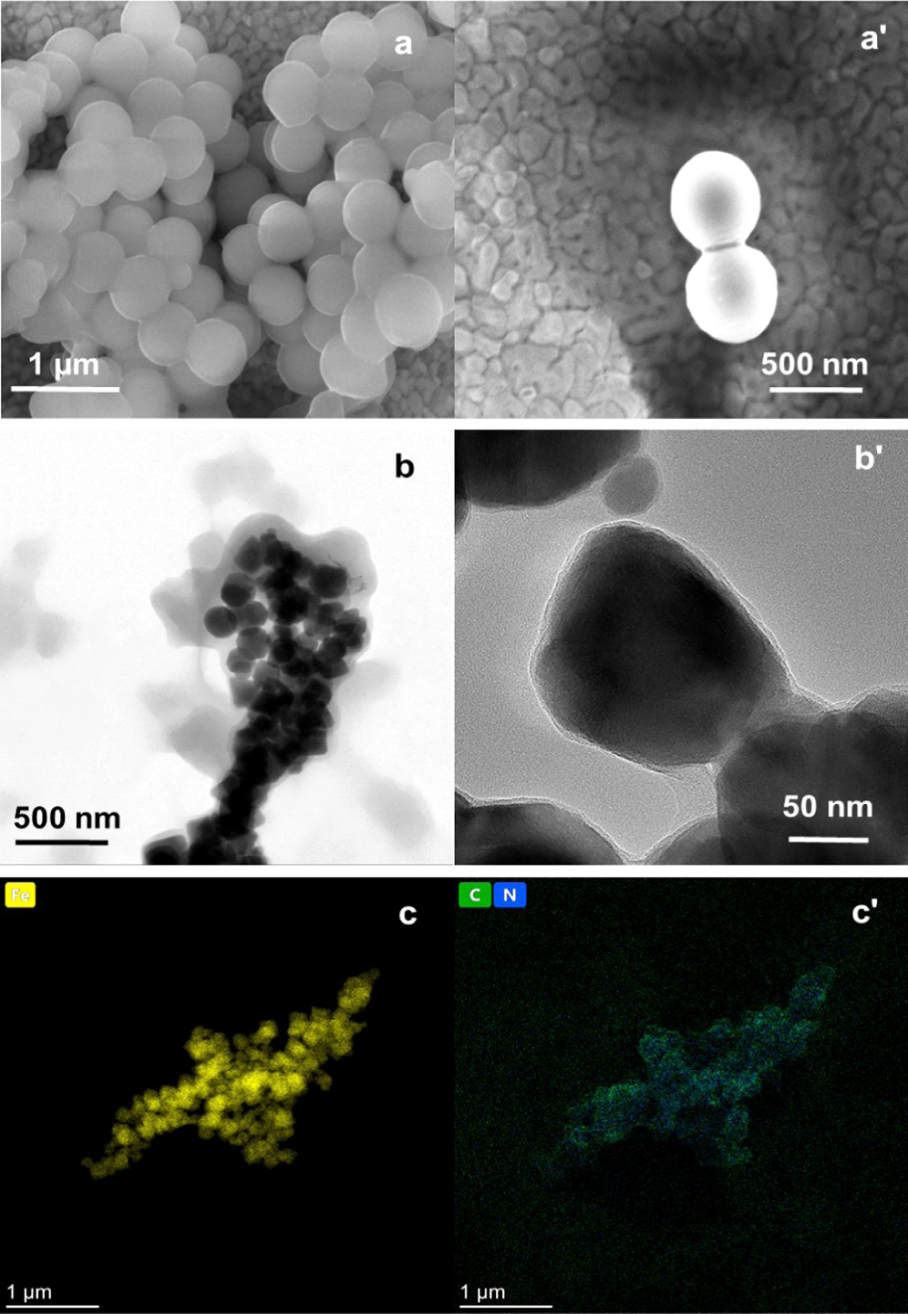
(a, a′) SEM images of the **NIPAM–MA–BIS**-coated SiO_2_ particles. (b, b′) TEM images of the **NIPAM–MA–BIS**-coated magnetic nanoparticles. (c, c′) Mapping elemental distribution of **NIPAM–MA–BIS** gel particles prepared using the **NIPAM, MA**, and **BIS** monomers of concentrations of 20, 20, and 10 mM, respectively.

Core–shell particles, such as **NIPAM–MA–BIS**-coated MNPs, were imaged to confirm the thin film coating. The image clearly shows that the gel films of the **NIPAM–MA–BIS** monomer combination coat the inorganic cores (Figure 2a,a′,b,b′). The TEM imaging confirms that the films are thin (Figure 2b,b′). Moreover, the EDX mapping supports this observation (Figure 2c,c′). This mapping revealed that the Fe core particle (yellow) was coated with a film containing carbon (green) and nitrogen (blue) elements.

### Structural Characterization of Gel Particles

FTIR spectroscopy was used to identify the functional groups in the **NIPAM–MA–BIS** nanogel particles (Figure S4 in the Supporting Information). The two bands at 2925 and 2980 cm^–1^ were observed accounted for the respective C–H bond’s asymmetric and symmetric stretching vibrations. The broad and intense band at 3327 cm^–1^ was associated with the N–H bond stretching. At 1388 and 1538 cm^–1^, the respective C–N (stretching vibration) and N–H (bending vibration) bands appeared. The split band at ∼1650 cm^–1^ corresponds to the amide I and amide II bands of **NIPAM** within the nanogel particles. The band at 1700 cm^–1^ is associated with the carbonyl group of **MA** in the nanogel particles. The sharp band positioned at 1160 cm^–1^ is assigned to the C–N bond vibration. These results confirm successful monomers’ copolymerization and gel formation.

### Dynamic Light Scattering (DLS) Study of Gel Particles in Solutions of Different pH Values

**Figure 3:**
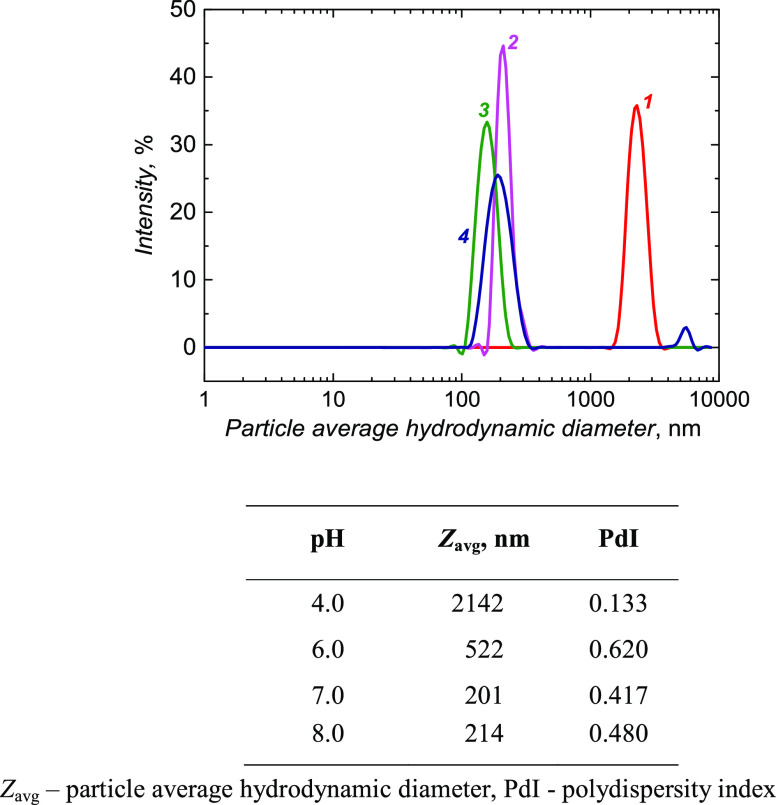
DLS analysis of **NIPAM–MA–BIS** gel nanoparticles dispersed in solutions of pH values (1) 4.0, (2) 6.0, (3) 7.0, and (4) 8.0.

Polyacrylamide nanogel particles’ properties are pH-dependent.^50−52^ For instance, the particles’ hydrophobic nature at low pH can be changed to hydrophilic at high pH in a phosphate buffer solution. The gel particles’ size decreased when the dispersion solution’s pH increased (Figure 3). The apparent p*K*_a_ of **MA** is 4.25. Therefore, at low pH, the protonated −COOH groups of **MA** molecules promote the aggregation of gel particles. However, at pH exceeding this p*K*_a_, the deprotonated −COO^–^ groups hinder gel aggregation because of Coulombic repulsion. Another possible explanation for the smaller gel size at an elevated pH could be the polymer chain opening and conforming into shorter globular chains. Furthermore, gel particles exhibited a random Brownian motion at all pH values. Their autocorrelation function’s decay is shown in Figure S5 in the Supporting Information.

### Biocompatibility Testing Using MTT Proliferation/Metabolic Activity Assay

**Figure 4:**
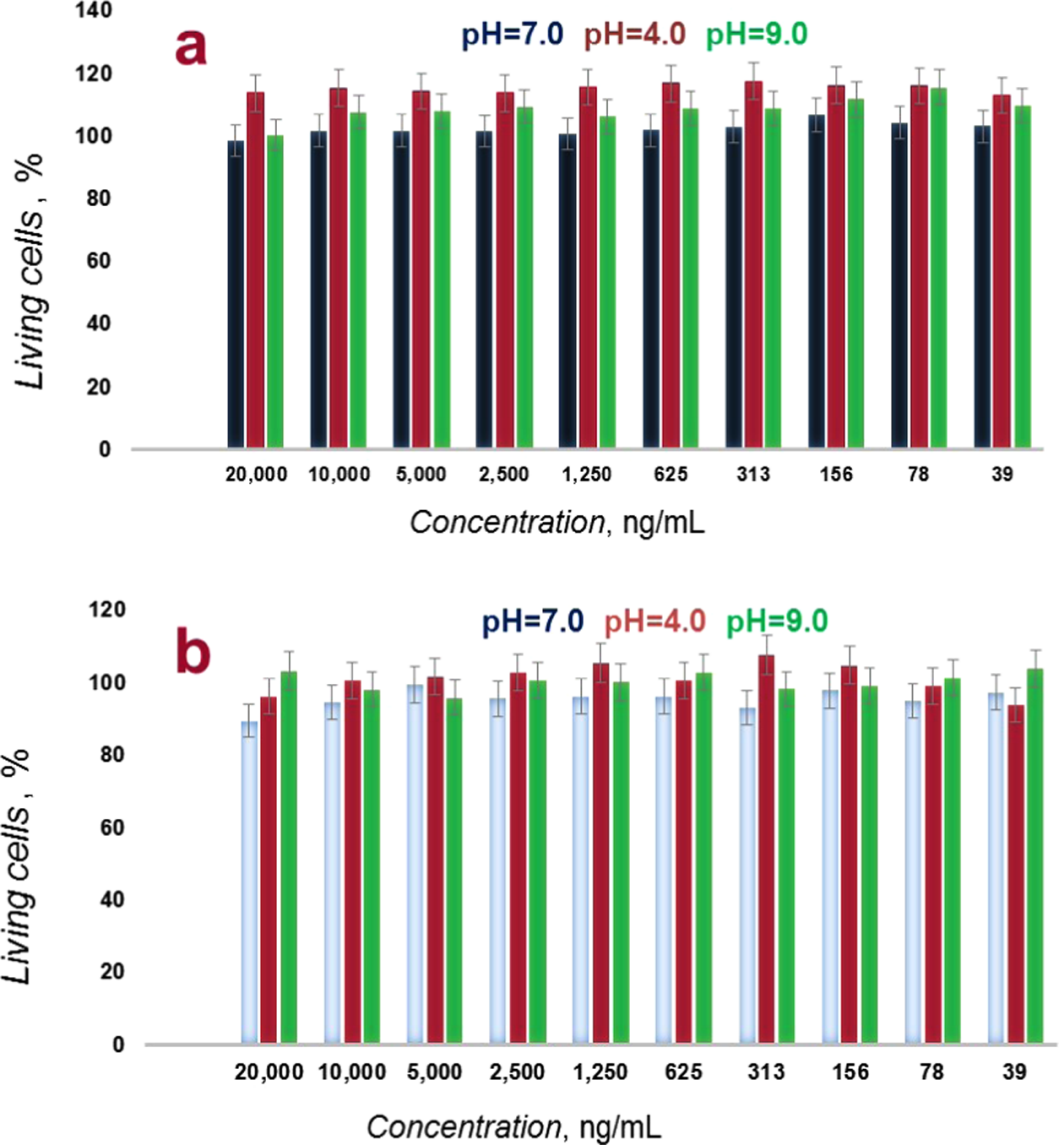
Histograms of MTT viability assay results of **NIPAM–MA–BIS** gel nanoparticles for (a) cervical cancer and (b) triple-negative breast cancer cell lines as a function of gel concentration. Different column colors refer to different pH values of the medium. Error bars correspond to standard deviations (*N* = 5).

For the prepared gel nanoparticles’ use as a 3D culture basement membrane-like ECM matrix, first, in vitro cytotoxicity assays were performed on two different cell lines, i.e., MDA-MB-231 (triple-negative breast cancer) and HeLa (cervical cancer), using the MTT proliferation/metabolic activity assay (Figure 4). Both cell lines were seeded in 96-well plates for 24 h before the experiment. Afterward, the dose-dependent cytotoxicity assay was performed for a specific time to determine the cell viability relative to the control. As the control, measurements were performed for untreated cells. The percentage of viable cells was calculated from the absorbance measurement data.

The IC_50_ concentration was determined from the plot of cell viability as a function of the nanoparticle concentration. Cell viability significantly decreased for the highest concentrations, namely, 33% of living cells for 1000 μg/mL (Figure S6 in the Supporting Information). The results agree well with literature data,^53,54^ indicating that the polyacrylamide gel is compatible with cells. An IC_50_ value of 485 μg/mL determined is only due to covering the surface available to the cells, resulting in a lack of access to oxygen and nutrients (Figure S7 in the Supporting Information). Two independent cytotoxicity assays were performed to test **NIPAM–MA–BIS** gel nanoparticles dispersed in acidic and basic solutions. Figure 4a,b shows cell viability as a function of the nanoparticles’ concentration. The results show that the **NIPAM–MA–BIS** gel nanoparticles dispersed in acidic and neutral solutions are nontoxic and highly biocompatible for both cell lines in a concentration range of 20,000–39 ng/mL.

### Long-Term Three-Dimensional (3D) Cell Culture

**Figure 5:**
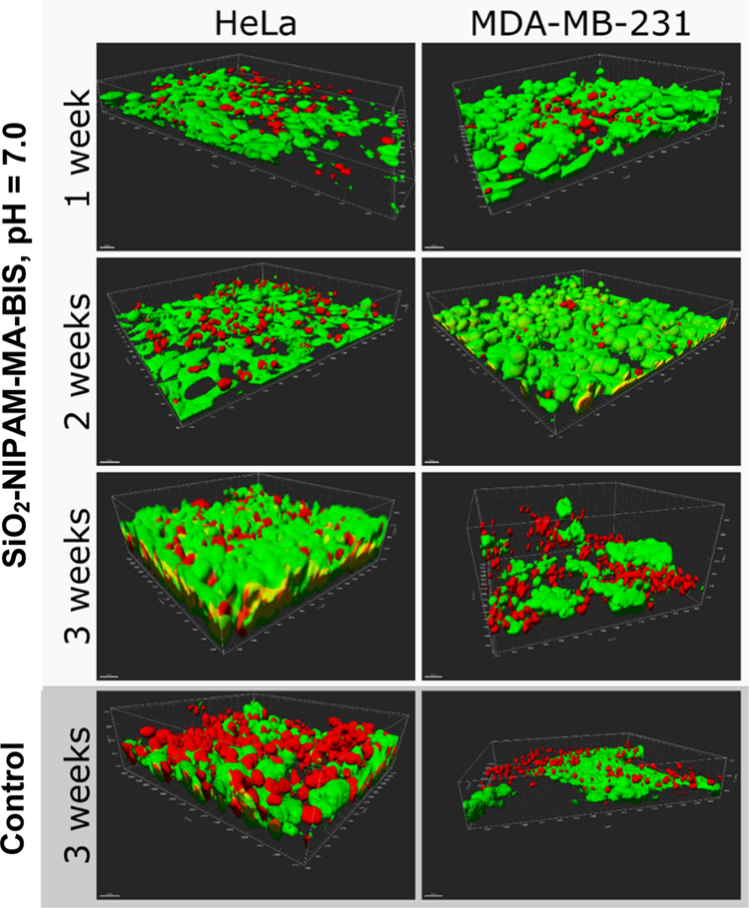
Confocal microscopy images of the long-term three-dimensional tissue culture on SiO_2_–**NIPAM–MA–BIS** core–shell particles at pH = 7.0. Green objects are viable cells stained with calcein-AM, while red objects are nuclei of dead cells stained with propidium iodide. Scale horizontal bars correspond to 30 μm.

Two model cell lines, HeLa and MDA-MB-231, were seeded with nanogels (SiO_2_–**NIPAM–MA–BIS**, MNP**–NIPAM–MA–BIS**, and **NIPAM–MA–BIS**) to check their usability for 3D cell cultures. The cultures were maintained for 3 weeks, with untreated cells seeded on the glass as the control. Half of the cell culture medium was replaced every second day (Figures 5 and S8–S10 in the Supporting Information). For each gel, two variants were considered, i.e., gel nanoparticles dispersed in solutions of pH values 4.0 and 7.0, and both were maintained at a neutral medium during the long-term cell culture. The glass support was chosen as a control to compare the spontaneous layered tissue formation of overconfluent culture with gel-assisted tissue formation.

3D confocal microscopy images of the cultures performed in the SiO_2_–**NIPAM–MA–BIS** gel are presented in Figures 5 and S10 in the Supporting Information. Similar data concerning MNP–**NIPAM–MA–BIS** and **NIPAM–MA–BIS**, at two pH values, are summarized in Figures S8 and S9 in the Supporting Information. Core–shell and gel nanoparticles supported the formation of thick and viable tissues of HeLa and MDA-MB-231 cells. Moreover, MDA-MB-231 cells formed complex 3D structures, where clusters of viable cells were surrounded by necrotic tissues. Moreover, the cell number and viability were significantly higher in the core–shell gel nanoparticle-supported cultures than in the controls.

**Figure 6:**
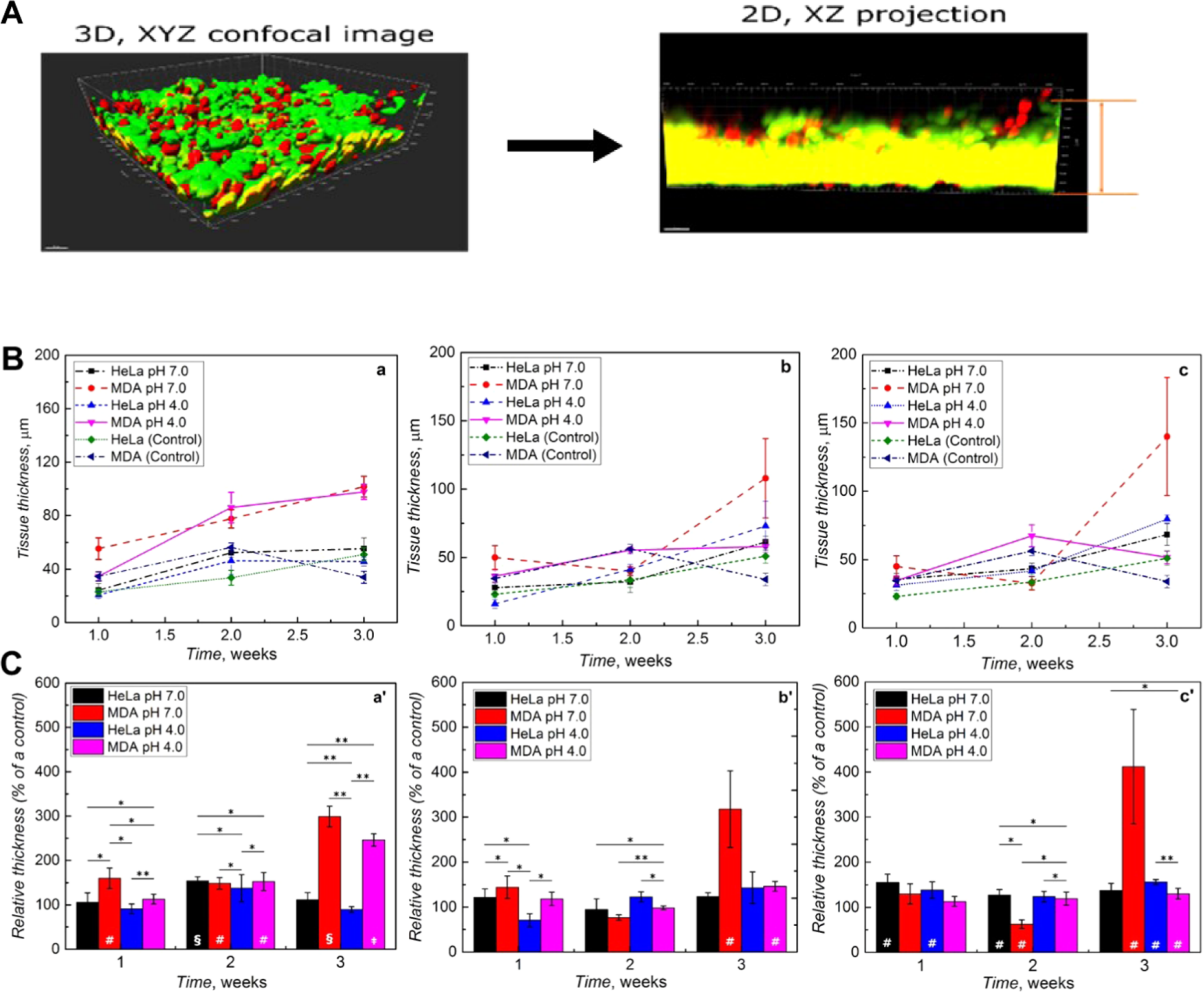
Quantitative analysis of tissue thickness growth during long-term cell culture in gels. (A) The measurement principle is illustrated by 3D confocal microscopy images processed to their 2D projections with Imaris software. Then, the thickness of the viable (green) tissue was measured from the 2D image. (B) Measurement results involving the absolute thickness of the cell cultures over gel nanoparticle comparison to the related controls: (a) **NIPAM–MA–BIS**, (b) SiO_2_–**NIPAM–MA–BIS**, and (c) MNP–**NIPAM–MA–BIS**. (C) The relative 3D cultures thickness changes with time, calculated as the fraction of the related controls (a′) **NIPAM–MA–BIS**, (b′) SiO_2_–**NIPAM–MA–BIS**, and (c′) MNP–**NIPAM–MA–BIS**. Error bars correspond to the standard deviation from three tissue slices. Statistical significance: single asterisks (*) denote significant statistical change (*P* < 0.05) and double (**) asterisks denote very significant (*P* < 0.005) statistical change between samples. Additionally, the statistical significance of the sample as compared to that of the control was marked within bars as follows: # for *P* < 0.05, § for *P* < 0.005, and ‡ for *P* < 0.0005.

The 3D XYZ images were processed to the 2D XZ or 2D YZ projections using Imaris software (Figure 6a) to obtain quantitative information on the tissue formation progress. Next, viable tissues’ thickness was determined from the 2D projections. These determinations’ results are presented in Figure 6b,c. The HeLa and MDA-MB-231 cells revealed a difference in the tissue growth dynamics (Figure 6b). During the first 2 weeks of culture, the control MDA-MB-231 cells without gel particles grew to form layers thicker than those formed by the HeLa cells. Massive degeneration and necrosis in MDA-MB-231 control cells were observed during the third week while HeLa cells continued growing (Figure 5).

On the other hand, the growth of gel-supported 3D cultures was proportional to that of the control in the case of HeLa cells. Gel-supported MDA-MB-231 cells developed structures much more complex and expended than the control (Figure 6a′). This development was particularly pronounced in the third culture week when the control degenerated (Figure 6a′). Presumably, gel nanoparticles support the extended 3D structure growth in the case of insufficient adhesion to the glass 2D supports. Moreover, the SiO_2_–**NIPAM–MA–BIS** (Figure 6b′) and MNP–**NIPAM–MA–BIS** core–shell nanogels (Figure 6c′) provided much more beneficial support for complex 3D tissue structures.

The glass-supported culture comparison with the gel-supported cultures leads to the following conclusion. At the initial stage of culture (the first week), there was no significant difference in culture morphology, suggesting no preference for the gel support over the glass support, and the cells formed a monolayer. However, gel-supported cultures grew thicker during subsequent weeks, while control cultures gradually degenerated. Seemingly, overconfluent cells adhered to the gel support, spread, and formed extensive 3D structures. Moreover, the nanogels’ porosity afforded sufficient oxygen and nutrient delivery, as viable cells were detected in the whole tissue (over 150 μm thick in the MNP–**NIPAM–MA–BIS** gel). On the other hand, overconfluent cells in glass controls underwent anoikis (apoptosis in response to inappropriate cell–support interactions). Manifesting core–shell gels are a promising tool for investigating more complex 3D cell cultures.

## Conclusions

We synthesized a series of **NIPAM–MA–BIS** gel nanoparticles without and with different inorganic cores. Surfaces of different cores were grafted with **NIPAM–MA–BIS** copolymer shells of the nanometer order thickness. Polyacrylamide was the major component of these shells. The gel nanoparticles’ morphology depended on the electroinitiation potential and the **BIS** cross-linking monomer concentration in the solution for polymerization. The cross-linking monomer concentration dominantly influenced gel particles’ morphology. Moreover, this concentration defined gel solubility. Low gel density helped incur the softness that further facilitated the ion diffusion. Therefore, gel nanoparticles could mimic the biological environment for tissue culture and exhibit biocompatibility. The **NIPAM–MA–BIS** gel nanoparticles support the extended 3D structure growth compared to the control. In all gel systems, SiO_2_–**NIPAM–MA–BIS** and MNP–**NIPAM–MA–BIS** core–shell gel nanoparticles provided support much more beneficial than the conventional glass support for complex 3D tissue structures.

## Supporting Information Available

Effect of initiator concentration and polymerization quencher; process in the vicinity of the electrode surface; SEM images of gels synthesized at different electroinitiation potentials after adding inhibitor and cross-linking monomer concentration; gel FTIR spectrum and correlation function intensity for nanogel particles at different pH values; MTT assay for the IC50 value; HeLa cell viability as a function of nanoparticles’ concentration; and confocal microscopy images of the long-term three-dimensional tissue culture on gel and core–shell particles (PDF)
