# Supplementary material for: Electrochemically Synthesized Polyacrylamide Gel and Core–Shell Nanoparticles for 3D Cell Culture Formation
Source: ACS Appl Mater Interfaces. 2022 Jul 18;14(29):32836–44. doi: 10.1021/acsami.2c04904 (PMC9335524; doi:10.1021/acsami.2c04904)
Supplement: Supplementary file 1 — am2c04904_si_001.pdf [file am2c04904_si_001.pdf]

Supporting Information

Electrochemically synthesized polyacrylamide gel and core-shell nanoparticles for 3D cell culture formation

Nabila Yasmeen,[a] *Aneta Karpinska,*[a] *Jakub Kalecki,*[a] *Wlodzimierz Kutner,*[a,b]

Karina Kwapiszewska,[a,]* *and Piyush Sindhu Sharma*[a,]*

[a] Institute of Physical Chemistry, Polish Academy of Sciences, Kasprzaka 44/52, 01-224 Warsaw, Poland

[b] Faculty of Mathematics and Natural Sciences. School of Sciences, Cardinal Stefan Wyszynski University in Warsaw, Wóycickiego 1/3, 01-938 Warsaw, Poland

Corresponding Authors

*E-mail: psharma@ichf.edu.pl (Piyush Sindhu Sharma)

*E-mail: kkwapiszewska@ichf.edu.pl (Karina Kwapiszewska)

S1. Effect of initiator concentration

A sufficient amount of peroxydisulfate initiator ions in the aqueous solution for polymerization is necessary to generate a sufficiently high number of active centers that could propagate to undergo a phase transition to form precursor particles or short oligomer chains. Monomers polymerized on these nuclei centers forming soft colloidal gel particles. Therefore, the amount of initiator in the solution was varied. A sufficient number of free radicals was generated in the solution for gel particle preparation at the initiator concentration as low as 15 mM.

S2. Effect of polymerization quencher

This radical polymerization might not be sufficiently quenched after seizing the electrochemical reaction. Therefore, a polymerization inhibitor, vis., hydroquinone monomethyl ether, was added after stopping electroinitiation. Three different inhibitor concentrations were used, i.e., 5, 15, and 25 mM, to study the polymerization chain inhibition effect on the gel particles' morphology (Figure S3). Nevertheless, globular gel particles of different sizes were obtained in each case.

S3. The process in the vicinity of the electrode surface

Electrochemically initiated *N*-isopropylacrylamide, methacrylic acid, and *N,N'*-methylenebisacrylamide polymerization is started by dissociating persulfate at the suitable potential. A pathway of persulfate reduction is proposed below. This pathway involves a direct electron transfer to the anion in the solution.

$$S_2O_8^{2-} + e^- \rightarrow SO_4^{2-} + SO_4^{\cdot -} \qquad \text{(S1a)}$$

$$SO_4^{\cdot -} + e^- \rightarrow SO_4^{2-} \qquad \text{(S1b)}$$

Similarly, as previously described,[1,2] with time, more and more ammonium persulfate is cleaved at the potential of -0.60 V (Equation S1a), and the amount of water-soluble free radicals becomes sufficient for initiating the polymerization. Kinetic studies of *N,N'*-methylenebisacrylamide and *N*-isopropyl acrylamide copolymerization indicated that the *N,N'*-methylenebisacrylamide monomer reacts faster than the hydrophilic *N*-isopropyl acrylamide monomer (Scheme S1). So in the first step, water-soluble free radicals attack the *N,N'*-methylenebisacrylamide monomer. The *N,N'*-methylenebisacrylamide redox-active center

later targets the *N*-isopropyl acrylamide and methacrylic acid monomer double bonds, resulting in more extended oligomers. Ultimately, these oligomers combine and nucleate to form gel particles.

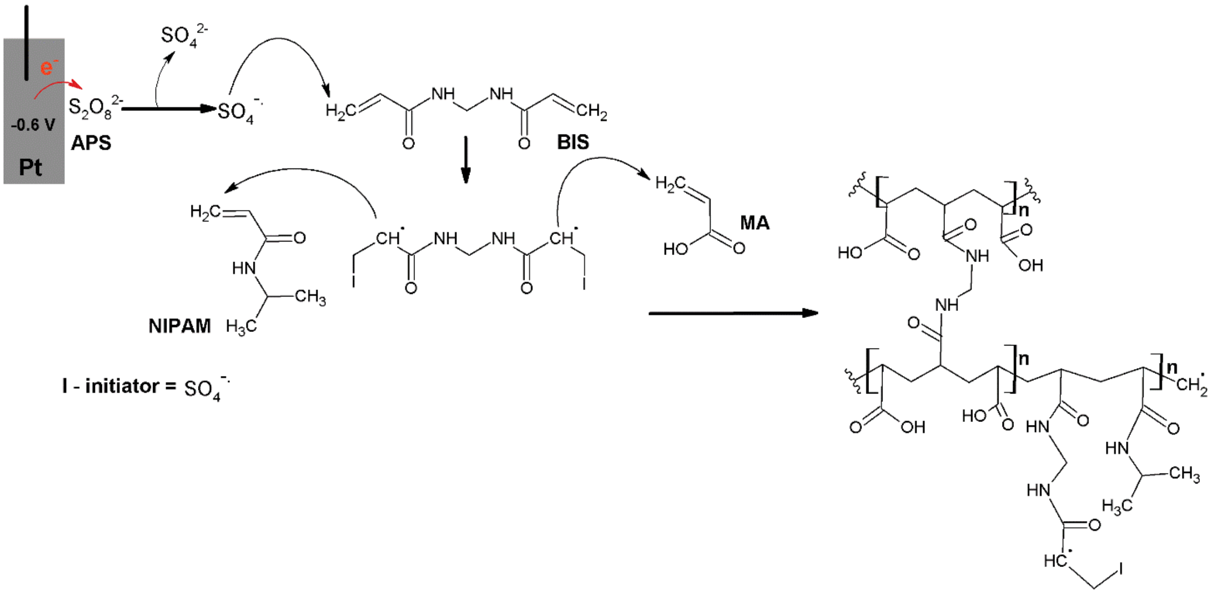

Scheme S1. The tentative mechanism of electrochemically synthesized gel nanoparticles.[1,2] **BIS** - *N*,*N'*-methylenebisacrylamide, **MA** - methacrylic acid, and **NIPAM** - *N*-isopropyl acrylamide.

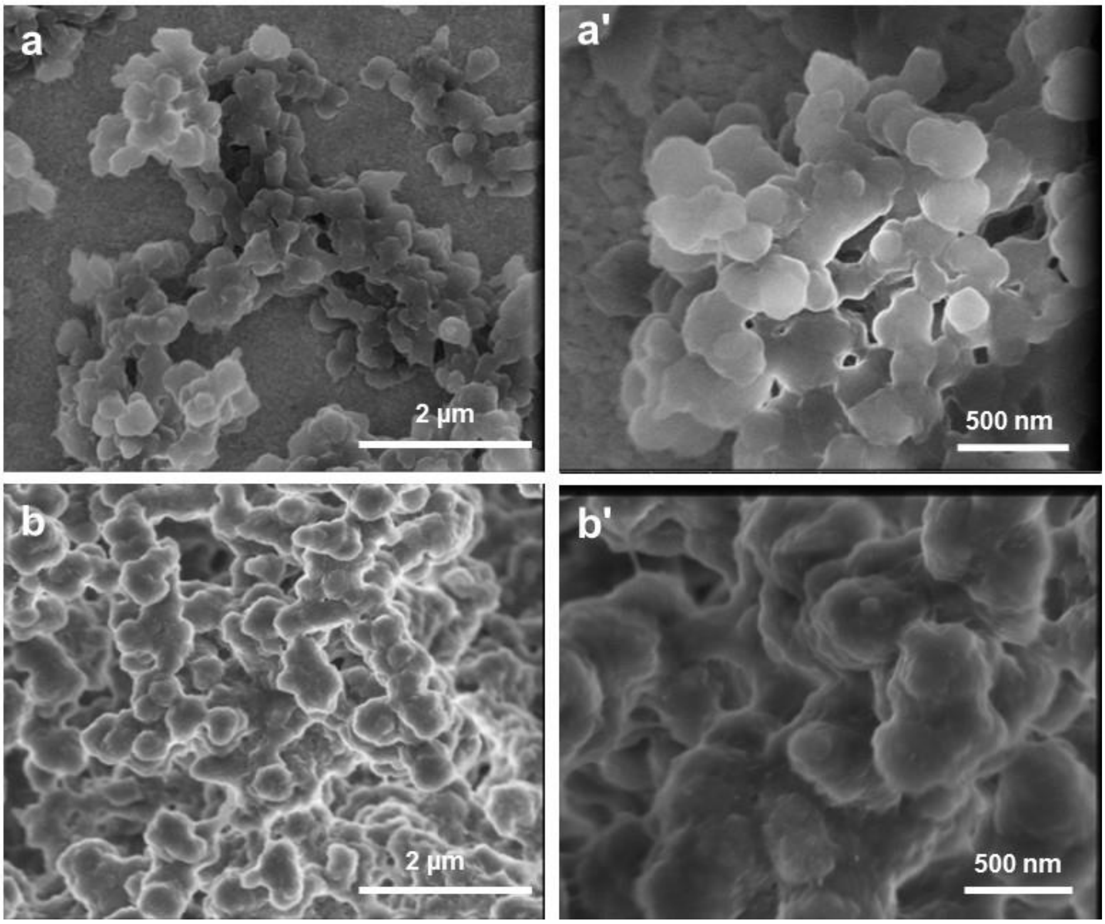

Figure S1. The SEM images of **NIPAM-MA-BIS** gels synthesized at the electroinitiation potential of (a, a') -0.70, and (b, b') -0.80 V vs. Ag quasi-reference electrode. The concentrations of **NIPAM, MA**, and **BIS** monomers in the solution for polymerization were 25, 25, and 50 mM, respectively.

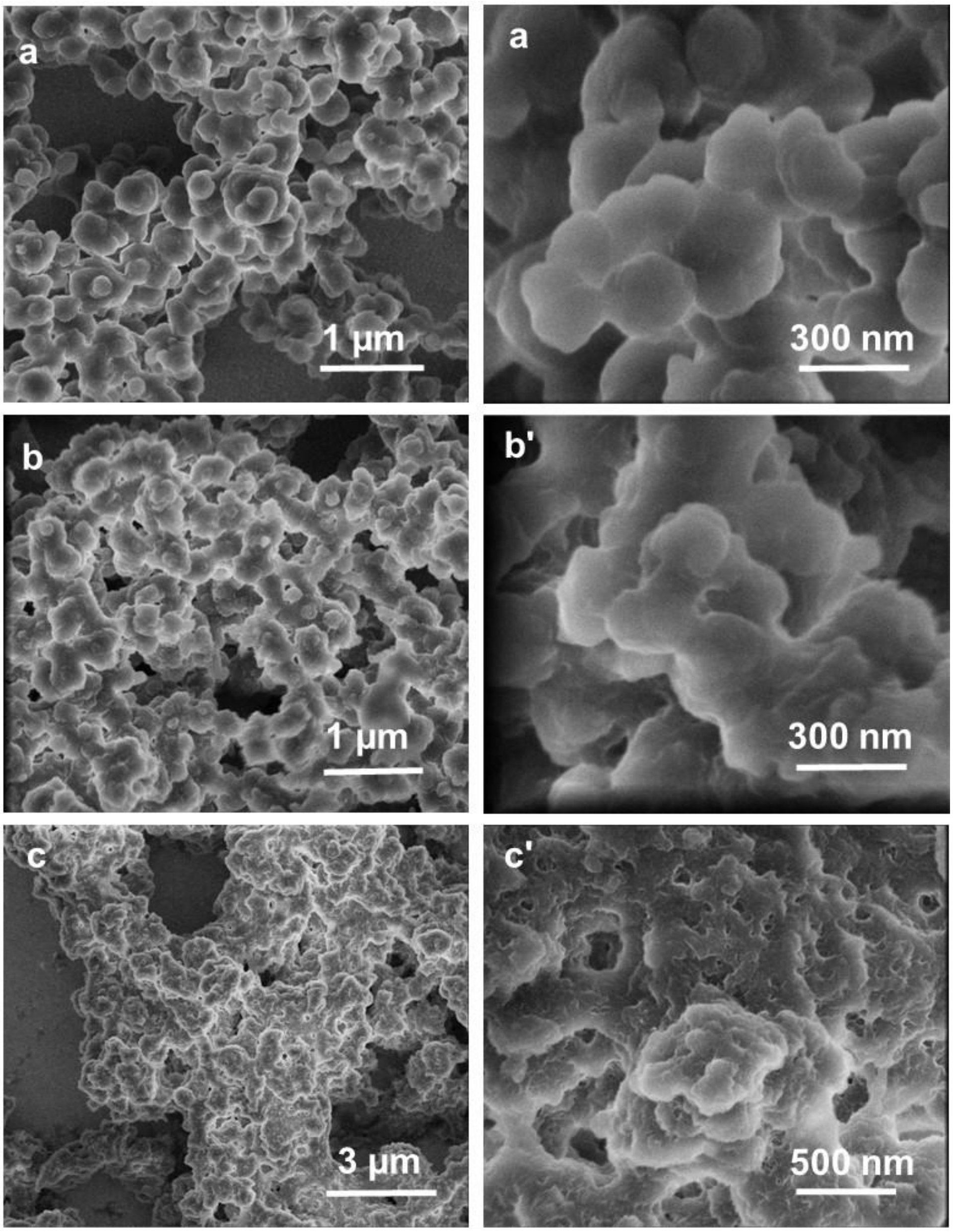

Figure S2. The **NIPAM-MA-BIS** gels' SEM images at the **BIS** cross-linking monomer concentration of (a, a') 5, (b, b') 50, and (c, c') 100 mM. Concentrations of the **NIPAM** and **MA** monomers in the solution for polymerization were 25 mM each.

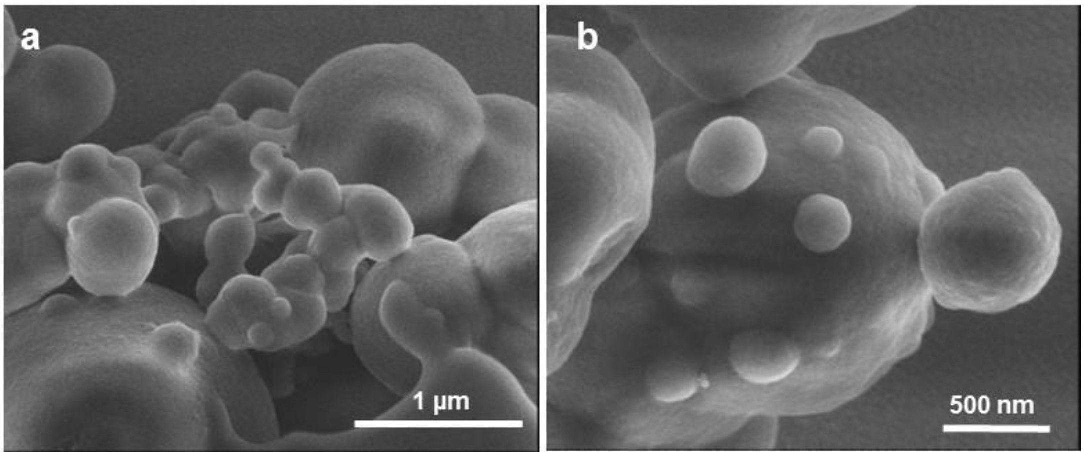

Figure S3. SEM images of gel particles prepared after inhibiting polymerization with 15 mM hydroquinone monomethyl ether. The gel was prepared by copolymerizing the **NIPAM**, **MA**, and **BIS** monomer at a 25, 25, and 50 mM concentration.

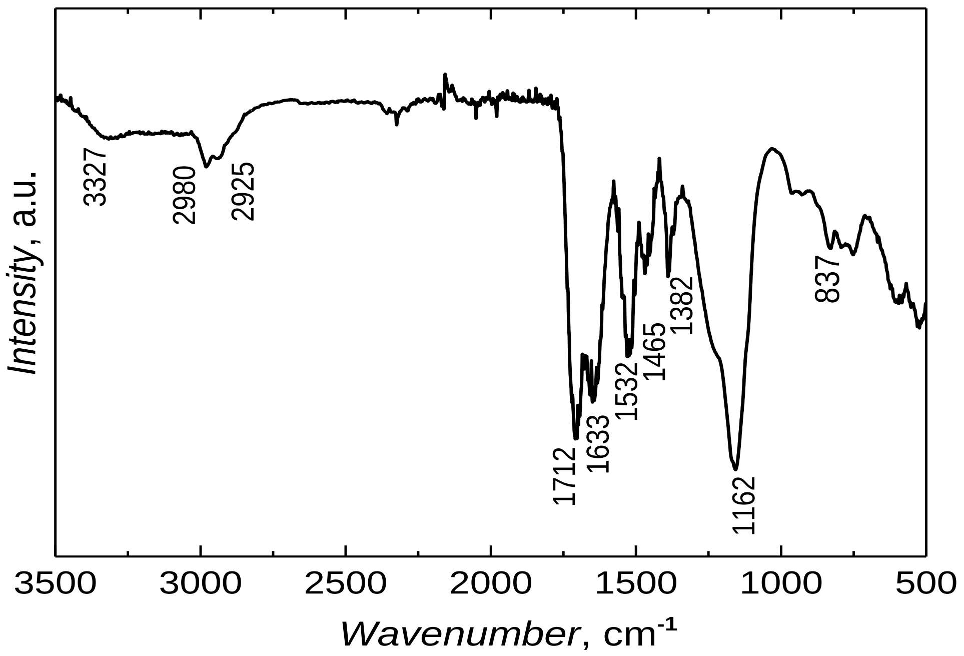

Figure S4. The FTIR transmission spectrum of the **NIPAM-MA-BIS** gel prepared by electrochemically initiated copolymerization of the **NIPAM, MA,** and **BIS** monomers of the concentration of 20, 20, and 10 mM, respectively.

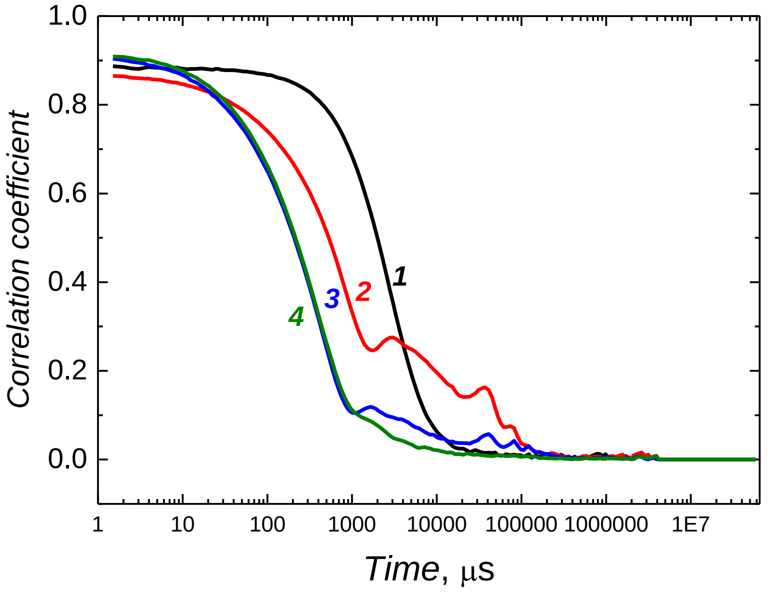

Figure S5. The correlation function intensity for **NIPAM-MA-BIS** nanogel particles in suspensions of pH of (*1*) 4.0, (*2*) 6.0, (*3*) 7.0, and (*4*) 8.0. The **NIPAM-MA-BIS** gel prepared by electrochemically initiated copolymerization of the **NIPAM, MA,** and **BIS** monomers of respective 20, 20, and 10 mM concentrations.

S4. MTT assay for IC_{50} value

The cell solution after the passage was diluted to an approximate number of ~10 000 cells/well (controlled with Countess II Cell Counter) and seeded into a 96-well plate (Greiner Bio-One). Then, cells were incubated for 24 h at 37 °C to enable cell attachment. The medium was removed, and **NIPAM-MA-BIS** gels were added to the fresh culture medium at different concentrations (1000 μg/mL to 2 μg/mL). We performed five repeats for each concentration. Moreover, controls were repeated five times: blank - medium without cells, positive control - cells not treated with the tested particles, and negative control - dead cells, toxicant - 1%, *v/v*, Triton-X 100 (Sigma-Aldrich). The plate was incubated at 37 °C for 24 hours. Afterward, the medium was replaced with a culture medium containing 1 mM 3-(4,5-dimethylthiazol-2-yl)-2,5-diphenyltetrazolium bromide (MTT reagent, Thermo Fischer Scientific). We incubated cells for 4 h at 37 °C. Then, solutions were replaced with DMSO and incubated for 10 min. The absorbance in each well was measured at 540 nm using a Synergy HTX multi-mode reader (BioTek). Two independent cytotoxicity assays were performed.

Data analysis

The percentage of viable cells in each sample compared to the control was calculated from the absorbance measurement data using Equation S3:

$$\text{Viability [\%]} = \frac{\text{Measured value} - \text{Average}_{\text{negative control}}}{\text{Average}_{\text{positive control}} - \text{Average}_{\text{negative control}}} \quad \text{(S3)}$$

Each data point averages five independent wells, and error bars correspond to the standard deviations. The IC_{50} concentration was determined from a plot of cell viability as a function of the nanoparticle concentration. Cell viability data were fitted with a Logistic Dose-Response model, with four fitting parameters (Equation S4)

$$y = \frac{A_1 - A_2}{1 + (x/IC_{50})^p} + A_2 \quad \text{(S4)}$$

where A1 and A2 were upper and lower asymptotes, respectively; the IC_{50} values were fitting parameters of the model. The fitting results are presented in Figures S6 and S7. The results obtained enabled determining the IC_{50} value as 485 μg/mL. The dose-response graph is shown in Figure S6 in the Supporting Information. The IC_{50} value corresponds to 15-fold coverage of the growth surface (Figure S7 in the Supporting Information). A significant decrease in cell

viability was observed for the highest concentrations (33% of living cells for 1 000 µg/ml). However, it is essential to remember that the IC_{50} value is closely related to the nanoparticles' coverage of the surface area available for cell growth.

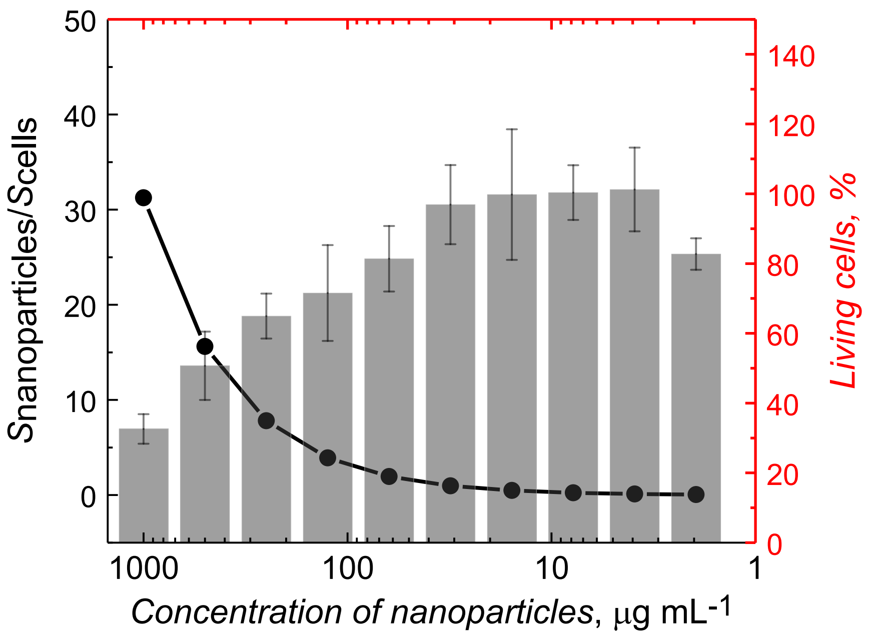

Figure S6. Gray column stands for HeLa cells viability as a function of nanoparticles' concentration. The black curve shows the ratio of the surface area occupied by nanoparticles to the surface area available to cells ($S_{\text{nanparticles}}/S_{\text{cells}}$).

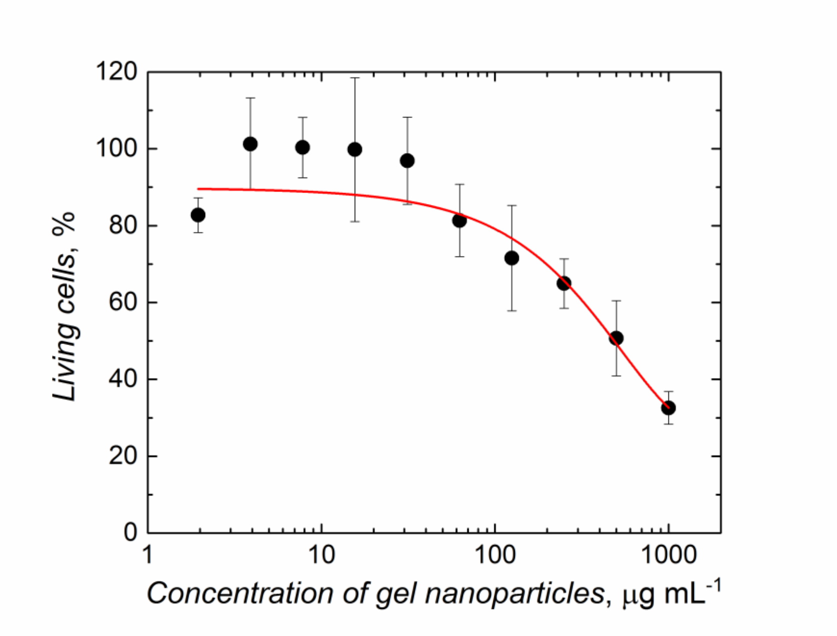

Figure S7. Viability of HeLa cells incubated for 24 h with **NIPAM-MA-BIS** gel nanoparticles as a function of gel nanoparticles' concentration, with the IC_{50} value (485 μg mL^{-1}) based on the MTT assay.

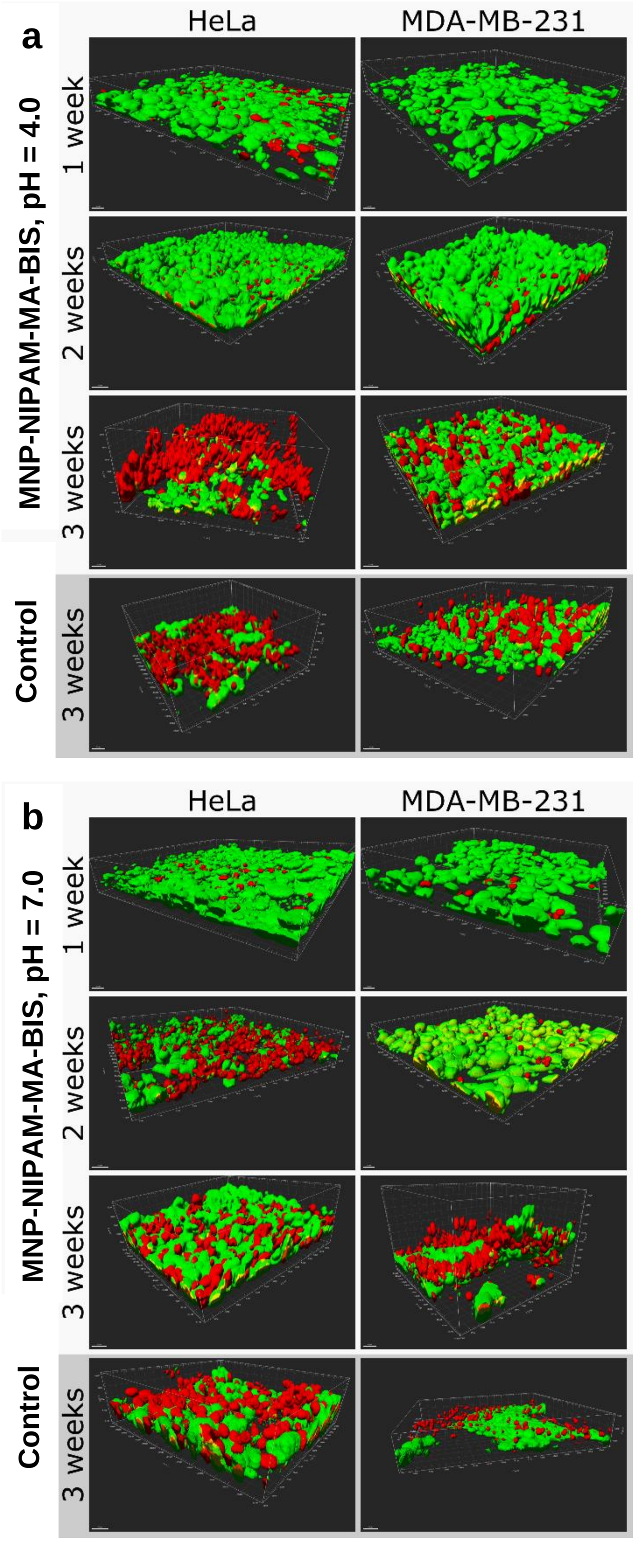

Figure S8. Confocal microscopy images of the long-term three-dimensional tissue culture on **MNP-NIPAM-MA-BIS** core-shell particles. Gel particles dispersed in solutions of pH of (a) 4.0 and (b) 7.0. Green objects are viable cells stained with calcein-AM, and red objects are nuclei of dead cells stained with propidium iodide. Scale horizontal bars correspond to 30 µm.

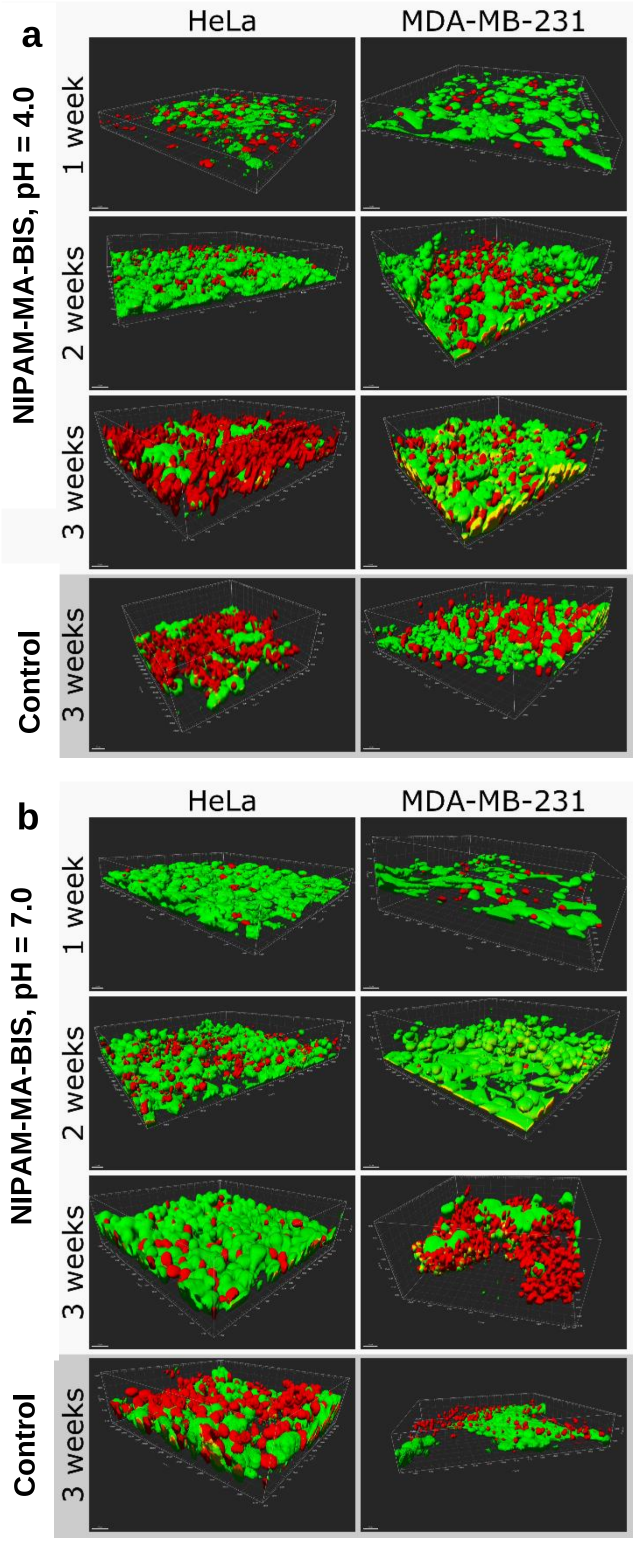

Figure S9. Confocal microscopy images of the long-term three-dimensional tissue culture on **NIPAM-MA-BIS** core-shell particles. Microgel particles dispersed in solutions of pH of (a) 4.0 and (b) 7.0. Green objects are viable cells stained with calcein-AM, and red objects are nuclei of dead cells stained with propidium iodide. Scale horizontal bars correspond to 30 µm.

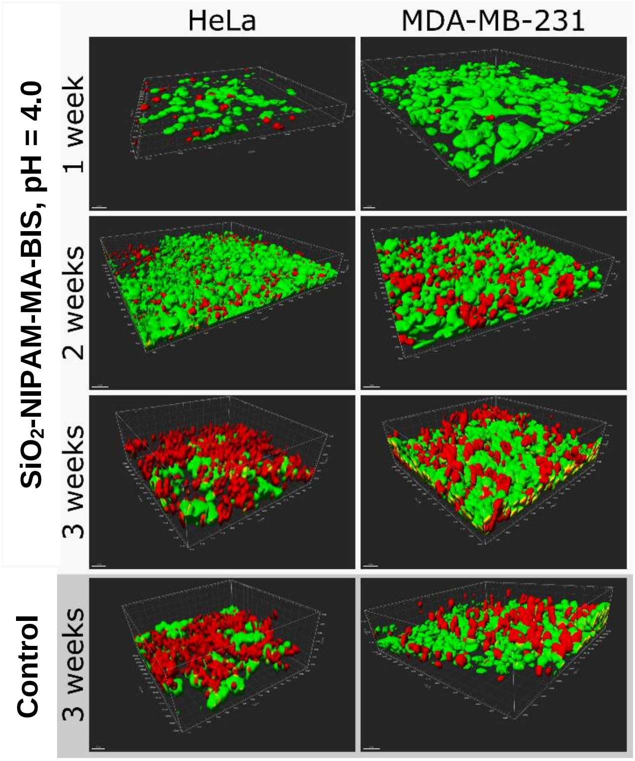

Figure S10. Confocal microscopy images of the long-term three-dimensional tissue culture on SiO_2-**NIPAM-MA-BIS** core-shell particles. Gel particles were dispersed in solutions of pH of (a) 4.0 and (b) 7.0. Green objects are viable cells stained with calcein-AM, and red objects are nuclei of dead cells stained with propidium iodide. Scale horizontal bars correspond to 30 µm.

REFERENCES

(1) Yasmeen, N.; Kalecki, J.; Borowicz, P.; Kutner, W.; Sharma, P. S. Electrochemically Initiated Synthesis of Polyacrylamide Microgels and Core-shell Particles. *ACS Appl. Polym. Mater.* **2022**, *4*, 452-462.

(2) Kaniewska, K.; Karbarz, M.; Stojek, Z. Electrochemical Attachment of Thermo- and pH Sensitive Interpenetrating-Polymers-Network Hydrogel to Conducting Surface. E*lectrochim. Acta* **2015**, *179*, 372-378.
